# A Metabolomic Landscape of Maize Plants Treated With a Microbial Biostimulant Under Well-Watered and Drought Conditions

**DOI:** 10.3389/fpls.2021.676632

**Published:** 2021-06-03

**Authors:** Lerato Nephali, Venessa Moodley, Lizelle Piater, Paul Steenkamp, Nombuso Buthelezi, Ian Dubery, Karl Burgess, Johan Huyser, Fidele Tugizimana

**Affiliations:** ^1^Department of Biochemistry, University of Johannesburg, Johannesburg, South Africa; ^2^International Research and Development Division, Omnia Group, Ltd., Johannesburg, South Africa; ^3^Institute of Quantitative Biology, Biochemistry and Biotechnology, School of Biological Sciences, University of Edinburgh, Edinburgh, United Kingdom

**Keywords:** plant biostimulants, PGPR, priming, metabolomics, maize, drought stress, microbial biostimulants

## Abstract

Microbial plant biostimulants have been successfully applied to improve plant growth, stress resilience and productivity. However, the mechanisms of action of biostimulants are still enigmatic, which is the main bottleneck for the fully realization and implementation of biostimulants into the agricultural industry. Here, we report the elucidation of a global metabolic landscape of maize (*Zea mays* L) leaves in response to a microbial biostimulant, under well-watered and drought conditions. The study reveals that the increased pool of tricarboxylic acid (TCA) intermediates, alterations in amino acid levels and differential changes in phenolics and lipids are key metabolic signatures induced by the application of the microbial-based biostimulant. These reconfigurations of metabolism gravitate toward growth-promotion and defense preconditioning of the plant. Furthermore, the application of microbial biostimulant conferred enhanced drought resilience to maize plants *via* altering key metabolic pathways involved in drought resistance mechanisms such as the redox homeostasis, strengthening of the plant cell wall, osmoregulation, energy production and membrane remodeling. For the first time, we show key molecular events, metabolic reprogramming, activated by a microbial biostimulant for plant growth promotion and defense priming. Thus, these elucidated metabolomic insights contribute to ongoing efforts in decoding modes of action of biostimulants and generating fundamental scientific knowledgebase that is necessary for the development of the plant biostimulants industry, for sustainable food security.

## Introduction

Drought stress is one of the major abiotic stresses constraining global crop production (Lesk et al., 2016). Further declines in crop production are expected in the near future, as exacerbated by unpredictable changes to global climates. Thus, urgent eco-friendly, sustainable and innovative strategies that can improve the crop production and tolerance to drought stress are imperatively needed to satisfy ever-increasing food demands (Reynolds et al., 2016). Emerging evidences have shown that plant biostimulants can function as plant priming agents, as demonstrated by the observed effectiveness of such biostimulants in promoting and sensitizing plant defenses and resistance against different environmental stresses, including drought (Fleming et al., 2019). The definitions and concepts of “biostimulants” have evolved overtime. Currently, a biostimulant is defined and described as a biological formulation that improves plant health and productivity as a resultant action induced by the novel, or emergent properties of the complex mixture, and not only from the presence of a plant growth regulator (Yakhin et al., 2017). The incorporation of biostimulant strategies and programs in the cropping system holds a promise to food sustainability. The biostimulant market is constantly on the economical rise due to the following: (i) the need to promote more efficient and effective use of organic materials for soil health sustainability, (ii) the growing availability of novel biostimulant formulations aiming at improving specific agronomic needs, and (iii) the unpredictable climate change leading to the increasing frequency of adverse environmental conditions negatively affecting the crop growth and productivity (Rouphael et al., 2020). Biostimulants are currently categorized into microbial and non-microbial formulations. A microbial-based biostimulant, which is the focus of this study, can be described as any formulation containing one or more microorganisms that has the ability to promote the health and growth of a plant through the induction of natural biological processes (du Jardin, 2015; Yakhin et al., 2017; Ricci et al., 2019).

Despite the advancements made in the last decade in terms of characterizing the effects of biostimulants on a wide range of plants, the modes of action of microbial biostimulants and the exact underlying biochemical and molecular events induced by these formulations to promote growth and enhance drought tolerance in plants remain enigmatic. This knowledge-gap hampers industries and famers from confidently and innovatively formulating and fully implementing the use of biostimulants into agronomic practices (Rouphael et al., 2020). Omics sciences, particularly metabolomics, are emerging as indispensable approaches in decoding the mechanisms of action employed by biostimulants (Nephali et al., 2020). Recent metabolomics studies have shown that a microbial biostimulant increased the yield of sweet pepper by inducing a metabolic readjustment of hormones and secondary metabolism (Bonini et al., 2020). Furthermore, some of the currently elucidated molecular changes underlying microbial-induced systemic resistance against drought stress include (i) the production of phytohormones, siderophores, volatiles and osmolytes, (ii) the enhanced antioxidant defense system and (iii) 1-aminocyclopropane-1-carboxylate (ACC) deaminase activity and exopolysaccharides (EPS) production (Ahemad and Kibret, 2014; Vurukonda et al., 2016; Prasad et al., 2017; Bhat et al., 2019). However, these reported events are a tip of an iceberg in regards to cellular and molecular phenomenology, metabolic profiles and fluxes that represent the integrated output of the molecular machinery and biochemical processes, activated by microbial biostimulants for plant growth promotion and stress resilience.

Thus, reported herein is a description of the metabolomic landscape that characterizes microbial biostimulant-induced mechanisms for growth promotion and improved drought tolerance in maize plants. Interrogating the metabolism of biostimulant-treated plants could reveal the metabolic plasticity and key metabolomic signatures that define the effects of microbial biostimulants on the plant physiology. Such insight provides a framework for mechanistic prediction and understanding microbial biostimulant-induced physiological changes in crop plants, under drought conditions; a key necessary step for the development of the plant biostimulants industry, for sustainable food security.

## Materials and Methods

All chemicals for sample analyses (from the pre-analytical step to the data acquisition stage) were of analytical or pure-grade quality and obtained from various international suppliers. Briefly, the organic solvents used, methanol and acetonitrile, were LC-MS grade quality (Romil, SPS, Cambridge, United Kingdom). Water was purified by a Milli-Q Gradient A10 system (Siemens, Fahrenburg, Germany). Leucine enkephalin and formic acid were purchased from Sigma Aldrich, Munich, Germany. The study design and plants’ cultivation are detailed in the following section.

### Plant Cultivation and Treatments

The microbial biostimulant formulation used in this study is BACSTIM^®^100 (Omnia Group Ltd., Bryanston, South Africa), a consortium of five *Bacilli* strains (viable PGPR): two strains of *Bacillus licheniformis*, two stains of *Brevibacillus laterosporus* and one strain of *Bacillus amyloliquefaciens*. This biostimulant formulation is a spore forming product, commercially tested for stability (Omnia Group Ltd., Bryanston, South Africa). In the following sections, for semantics simplicity, the expressions microbial biostimulant, biostimulant and PGPR are used interchangeably to refer to this biostimulant product, BACSTIM^®^100. The study was experimentally designed to comprise control (C), a group of plants with no stress and no PGPR applications and treated groups included P/PGPR (no stress applied but with PGPR treatment), DS (drought stress application but no PGPR treatment) and DS + PGPR (drought stress application and PGPR treatment). Each pot was considered as a biological replicate and contained five plants at the harvesting time. Three biological replicates (i.e., three pots) per treatment were harvested at each time point.

The maize (*Zea mays*) plants, PAN 3Q-240, were cultivated in 10 L pots filled with 17 kg of sandy soil (with pH of 4.6, bulk density of 1,495 kg.m^–3^, organic carbon of 0.22% m/m and organic matter of 0.38% m/m) in a greenhouse at Omnia facilities in Sasolburg, Free-State, South Africa. For nutrition, a fertilizer band containing 30 kg N/ha, 30 kg P/ha, and 30 kg K/ha was placed in the middle of each pot, five maize seeds were then placed in a row 5 cm to either side of the band at 3 cm depth. After emergence, thinning was applied, 5 uniform and healthy plants were selected per pot. At 3-leaf stage (3 weeks after emergence, WAE), all plants were top-dressed with 30 kg N/ha. The microbial (biostimulant) treatment was applied at planting at the application rate of 2 L/ha in soil furrow with a seed. All pots were irrigated to 90% plant available water (PAW) to allow for good germination. Drought stress was imposed at the 3-leaf stage (3 WAE): the water level was allowed to drop to 50% PAW and was maintained at that level for the drought-stressed group of plants. The well-watered plants maintained the 90% PAW throughout the experiment. The 50% PAW and 90% PAW were calculated using the equations provided in Supplementary File (section S2.1).

### Harvesting Plant Materials and Metabolite Extraction

Harvesting of the plant leaves was done a week after drought application and carried out at four different time points (4-, 5-, 6-, and 7 WAE) for all treatments and biological replicates. Metabolites were extracted by adding liquid nitrogen to the plant leaves followed by grinding of the frozen material to a powder form using pestle and mortar. Two grams (2 g) of the powder and 20 mL of 80% cold methanol was added in a 1:10 m/v ratio. The mixture was then homogenized for 2 min using Ultra-Turrax homogenizer and sonicated for 30 s using a probe sonicator (Bandelin Sonopuls, Germany) set at 55% power. The homogenates were centrifuged at 5,100 rpm for 20 min at 4°C. The supernatants were evaporated to 1 mL at 55°C using a Büchi Rotavapor R-200, and dried to completeness with a speed vacuum concentrator (Eppendorf, Merck, South Africa) set at 45°C. The dried material was re-suspended in 500 μL 50% LC-MS grade methanol and filtered into HPLC glass vials (Shimadzu, South Africa). The quality control (QC) samples were pooled from all samples.

### Data Acquisition Using Ultra-High-Performance Liquid Chromatography–High Definition Mass Spectrometry (UHPLC-HDMS) for Untargeted Metabolomics

The aqueous-methanol extracts were analyzed using a UHPLC-MS analytical platform. The chromatography was performed on an Acquity UHPLC system (Waters Corporations, Milford, United States) using a conditioned autosampler at 4°C. Two microliter of the sample was injected into the UHPLC system and chromatographically separated on a Waters analytical C18 column, HSS T3 (1.8 μm, 2.1 × 150 mm) thermostatted at 60°C, a reverse-phase column. The elution gradient was carried out with a binary (degassed) solvent system consisting of 0.1% aqueous formic acid (solvent A) and 0.1% formic acid in acetonitrile (solvent B) at a constant flow rate of 0.4 mL min^–1^. The initial conditions (98% solvent A and 2% solvent B) were held for 1 min. The conditions were then gradually changed to 30% solvent A and 70% solvent B at 14 min, followed by a change at 15 min to 5% solvent A and 95% solvent B which were held for 2 min and then changed to the initial conditions at 18 min. The analytical column was allowed to calibrate for 2 min before the next injection. The total chromatographic run time was 20 min.

The chromatography (LC) system was interfaced in-line with a high-definition MS platform, a SYNAPT G1 Q-TOF mass spectrometer (Waters Corporation, Milford, United States), equipped with electrospray ionization (ESI) source, for MS analyses. The time-of-flight (TOF) analyzer was operated in V-optics mode. The centroid data were acquired in both ESI positive and negative modes with the scan range of 50–1,200 Da, with a scan time of 0.1 s and an inter-scan delay of 0.02 s. The MassLynx^TM^ software automatically corrects the centroid mass values using leucine enkephalin as a reference calibrant in the sample for small deviations from the exact mass measurement, giving typical mass accuracies between 1 and 3 mDa. The other MS conditions for analyses were as follows: source temperature at 120°C, desolvation temperature at 450°C, capillary voltage 2.5 kV, sampling cone at 30 V, extraction cone at 4 V, cone gas flow 50 Lh^–1^, desolvation gas flow at 550 Lh^–1^. To generate molecular fragment information for downstream structure elucidation and compound identification, a data-independent acquisition (DIA) method, namely MS^*E*^ (also called MS^*All*^), was applied: the MS analyses were set to carry out non-fragmented as well as five fragmenting experiments simultaneously by applying alternating collision energy of 0 eV (unfragmented) and from 10 to 50 eV (fragmented). The software used to control the hyphenated system and perform all data manipulation was MassLynx^TM^ 4.1 (SCN 704, Waters Corporation Milford, United States). To assess the reliability and reproducibility of the analyses, and for non-linear signal correction, the QC samples were used. The blank samples (50% aqueous methanol) were also randomly run to monitor background noise.

### Data Acquisition on the LC-ESI-QqQ-MS System for Targeted Metabolomics

Amino acid and phytohormone standards used in this study were ≥98% purity and obtained from three suppliers: Merck (Germany), Sigma (United States of America) and BDH (England). The mixed working solutions of the amino acids, over the concentration range of 25–1,000 μg/L were prepared in 50% aqueous methanol (Romil, Cambridge, United Kingdom) and stored at 4°C. The concentration range of working solutions of phytohormones was 8.7×^–5^ – 43.7 nM prepared in 50% aqueous methanol and stored also at 4°C. The triple quadrupole mass spectrometry platform used was an LCMS-8050 (Shimadzu, Kyoto, Japan), equipped with an electrospray ionization (ESI) source and ultra-fast liquid chromatography (UFLC) as a front-end. The multiple reaction monitoring (MRM) method was used for absolute quantification of the targeted metabolites. The MRM-MS conditions were developed and optimized by direct infusion (using ESI source of MS); and the collision energy (CE) was optimized for each transition using the “MRM optimization method tool,” an integral component of LabSolutions LCMS software (Shimadzu Corporation). The tool automates the process by collecting product ion scan data and finding the optimum CE for each transition. These MRM optimal conditions are reported in Supplementary Table 1.

Both samples and working solutions of standards were analyzed on ultra-fast liquid chromatography (UFLC) system, fitted with Shim-pack GIST C18 column (2 μm; 100 × 2.1 mm l.D) (Shimadzu, Kyoto, Japan), thermostatted at 40°C. Chromatographic separation was performed using gradient elution utilizing eluent A (MilliQ water with 0.1% formic acid) and eluent B (methanol with 0.1% formic acid) (Romil Chemistry, United Kingdom) at a constant flow rate of 0.2 mL min^–1^. Different gradient elution methods were used for amino acid and hormone analysis, respectively. The gradient elution used for amino acids was as follows: the initial conditions of 98% solvent A and 2% solvent B were held for 1 min. From 1 to 3 min the conditions were changed to 95% solvent A and 5% solvent B, followed by a change at 3 min to 90% solvent A and 10% solvent B which were held for 2 min and then changed to 50% solvent A and 50% solvent B which was held for 2 min, then changed to the initial conditions. The total chromatographic run time was 10 min and the injection volume was 3 μL. The gradient elution used for hormone analyses was as follows: 98% solvent A and 2% solvent B over 0–3 min, 90% solvent A and 10% solvent B over 3–6 min, 80% solvent A and 20% solvent B held over 6–30 min and 95% solvent A and 5% solvent B over 30–38 min, 98% solvent A and 2% solvent B over 38–40 min. The total chromatographic run time for hormone analyses was 40 min and the injection volume was 1 μL. For MS analyses, the MRM-MS method was developed, optimized and applied (Supplementary Table 1). The MS conditions were as follows: nitrogen gas was used as a drying gas at 10 L min^–1^ flow rate and as a nebulizing gas at 3 L min^–1^ flow rate, the heating gas flow was set at 10 L min^–1^, interface temperature was set at 300°C, interface voltage was set at 4 kV, DL temperature was set at 250°C, heat block temperature was set at 400°C.

### Data Mining: Data Processing and Multivariate Data Exploration

Both centroid ESI positive and negative raw data from UHPLC-HDMS analyses were analyzed. Data visualization and processing were performed using MassLynx XS^TM^ 4.1 software (Waters Corporation, Manchester, United Kingdom). The MarkerLynx^TM^ application manager of the MassLynx software was used for data pre-processing, producing a data matrix of retention time (Rt)-*m/z* variable pairs, with *m/z* peak intensity for each sample. The parameters of the MarkerLynx application were set to analyze the 0.8–15 min Rt range (for both ESI positive and negative data) of the mass chromatograms, *m/z* domain of mass range 100–1,000 Da, with a mass tolerance of 0.05 Da and intensity threshold of 100, for both ESI positive and negative data. For the alignment of peaks across samples, the Rts were allowed to differ by ± 0.2 min (for ESI positive data), ± 0.3 min (for ESI negative data) and the *m/z* values by ± 0.05 Da. Furthermore, normalization was done by using total ion intensities of each defined peak; and prior to computing intensities. The generated data matrices (cleaned data, with its complex covariance structure) were exported into SIMCA (soft independent modeling of class analogy) software, version 15 (Umetrics, Umeå, Sweden) for data mining. The approach opted for in this study was to firstly explore the data using unsupervised methods, such as principal component analysis (PCA), following unsupervised modeling, such as orthogonal partial least squares-discriminant analysis (OPLS-DA). Different metrics and tests were applied for model validation and these include evaluation of explained and predicted variation (cumulative R^2^ and Q^2^), receiver operator classifier (ROC), cross-validation analysis of variance (CV-ANOVA) and permutation testing. MetaboAnalyst v 4.0^1^ was also used for further statistical analyses where necessary, mainly for the targeted analysis. This includes PLS-DA and generation of heatmaps for quantitative description of the results.

### Metabolite Annotation and Biological Interpretation

The metabolite features were annotated through a multistep workflow; and metabolites were annotated to the level 2 as classified by the Metabolomics Standard Initiative (MSI) (Sumner et al., 2007). Thus, the main steps followed for metabolite annotation in this study included: (i) deriving the molecular formula (MF) from full-scan accurate mass data; and chemical as well as heuristic rules (incorporated in MarkerLynx formula generator algorithm), such as mass differences, nitrogen rules, restrictions of element numbers, isotopic fit and rings-and-double-bond equivalent (Kind and Fiehn, 2007; Nash and Dunn, 2018) were used to filter MF obtained from accurate mass measurements; (ii) the selected MF was manually searched against databases and bioinformatics tools mainly, Dictionary of Natural Product (DNP)^2^, Chemspider^3^, PlantCyc^4^ to putatively assign compound names to the MF; (iii) structural elucidation was done through careful inspection of fragmentation patterns (structural fingerprints) by examining the MS^1^ and MS^*E*^ spectra of the selected metabolite candidate; (iv) structural confirmation was done by comparative assessment of *in-silico* and experimental fragmentation information, searching against in-house spectral library and annotation details (of a metabolite under consideration) reported in the literature. Only molecular structures with confirmed diagnostic fragments (structural fingerprints) were retained.

All annotated and targeted metabolites were used for metabolic pathway analysis, performed with the MetPA (Metabolomics Pathway Analysis) component of the MetaboAnalyst bioinformatics tool suite (version 4.0)^5^. Pathway analysis enables the identification of the affected metabolic pathways, analysis thereof and visualization. MetPA uses high-quality KEGG metabolic pathways as the backend knowledge base. In addition to existing literature, the use of these bioinformatics tools (for pathway analysis) provided a framework to partially map the molecular landscape of the metabolism understudy, enabling the biological interpretability of observed changes in a metabolome view.

## Results and Discussion

The microbial biostimulant used in this study was a consortium of five *Bacillus* strains, referred hereafter as simply plant growth-promoting rhizobacteria (PGPR). Methanol extracts of maize leaves were analyzed *via* LC-MS and different chemometrics methods and bioinformatics tools were applied to mine and interpret the spectral data (see the experimental section). Differential chromatographic profiles were observed (Supplementary Figure 1), reflecting treatment-related changes in the extracted metabolome. Principal component analysis (PCA) revealed key characteristics of the data, which include both drought- and PGPR-related sample grouping (Supplementary Figures 2A, 3A).

**FIGURE 1:**
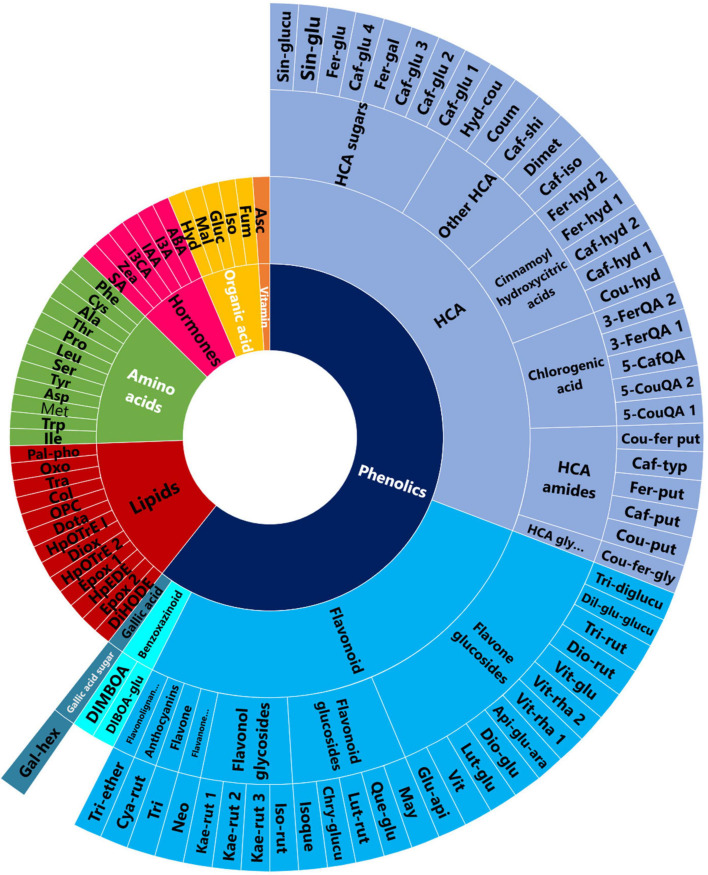
PGPR-induced metabolic changes spanned various classes of metabolites. A sunburst plot showing the classification of all the putatively annotated metabolites, and amino acids and hormones which were identified using targeted analysis. Refer to Supplementary Tables 1, 2 for the full names of the metabolites shown in this sunburst plot.

Sample classification modeling was performed using a multivariate hybrid machine learning method, orthogonal partial least squares-discriminant analysis (OPLS-DA). The generated OPLS-DA models showed treatment-related sample classifications and aided in extracting differential metabolite features (Supplementary Figures 2B,C, 3B,C and Supplementary Table 1). The PGPR-induced metabolic alterations spanned a wide spectrum of metabolite classes (Figure 1) including phenolic compounds, lipids, organic acids, hormones and amino acids. To epistemologically articulate these differential changes in the extracted maize metabolomes, two main aspects are pointed out, postulating thus a hypothetical framework that describes PGPR-induced reprogramming of maize metabolism toward (i) growth promotion and cellular priming, and (ii) a translation into enhanced resistance against abiotic stress, drought conditions in this case.

### Metabolic Reconfigurations Related to the Microbial Biostimulant-Induced Growth Promotion and Priming Effects

**FIGURE 2:**
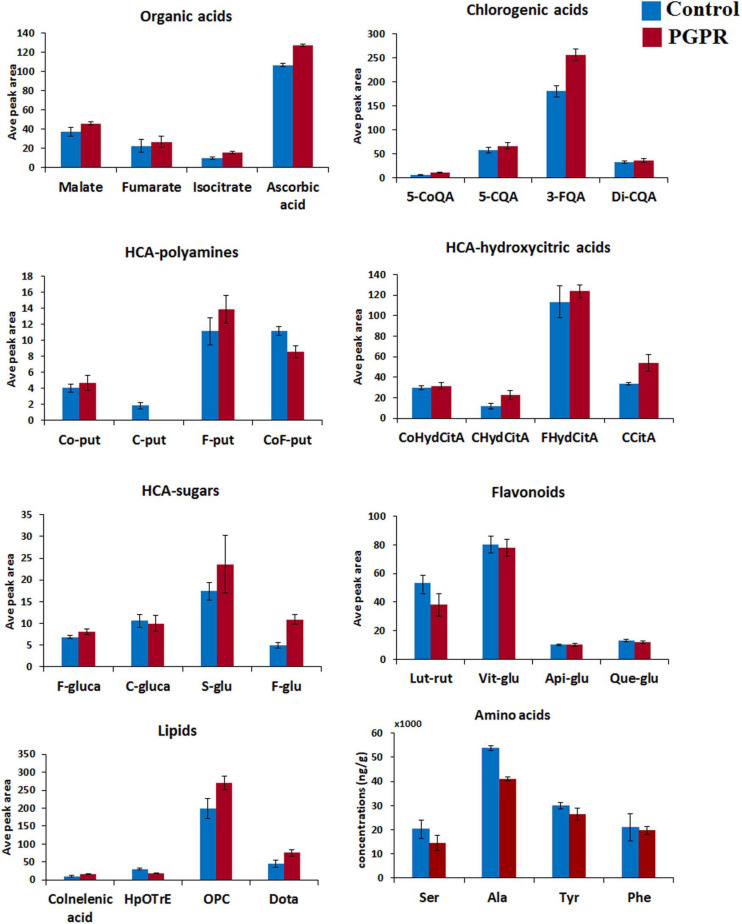
Differential quantitative profiles of some of the representative metabolites within main classes (organic acids, chlorogenic acids, HCA-hydroxycitric acids, HCA-amides, flavonoids, amino acids and lipids) in maize leaves. The bar graphs were generated using the average integrated peak area (*n* = 9) and the error bars represent the standard deviation. For statistical significance evaluation, the *p*-values are reported Supplementary Table 1. CoQA, coumaroylquinic acid; CQA, caffeoylqunic acid; FQA, feruloylquinic acid; Co-put, coumaroyl putrescine; C-put, caffeoyl putrescine; F-put, feruloyl putrescine; CoF-put, coumaroyl feruloyl putrescine; CoHydCitA, coumaroyl hydroxycitric acid; CHydCitA, caffeoyl hydroxycitric acid; FHydCitA, feruloyl hydroxycitric acid; CCitA, caffeoylisocitric acid; F-gluca, feruloyl glucarate; C-gluca, caffeoyl glucarate; S-glu, sinapoyl glucose; F-glu, feruloyl glucose; Lut-rut, luteolin rutinoside; Vit-glu, vitexin; Api-glu, apigenin glucose; Que-glu, quercetin glucose; Dota, dihydroxy-octadecatrienoic acid; OPC, oxo-(pentenyl)cyclopentane-octanoic acid; HpOTrE, hydroperoxy-octadecatrienoic acid; Ser, serine; Ala, alanine; Tyr, tyrosinel; Phe, phenylalanine.

The application of PGPR-based biostimulant on maize plants under normal conditions induced a global reprogramming of primary and secondary metabolism (Figure 1 and Supplementary Table 1). Primary metabolism is known to be directly involved in the normal plant growth and development. In this study, we observed decreased levels of alanine, serine, aspartic acid, cysteine, proline and threonine in PGPR-treated (non-stressed) plants (Figure 2 and Supplementary Figure 4). These differential changes in amino acid levels suggest a metabolic rewiring toward energy production; considering that these amino acids can be catabolized into upstream precursors of tricarboxylic acid (TCA) cycle compounds or intermediates (Hildebrandt et al., 2015). This correlates also to the measured accumulation of the TCA cycle intermediates such as fumarate, isocitrate and malate in PGPR-treated plants as compared to the naïve plants (Figure 2). Moreover, as building blocks of proteins, the decreased levels of amino acids, may point further to the increased rates of protein synthesis; which correlates to the measured higher aboveground dry-biomass of PGPR-treated maize plants compared to non-treated plants, under normal conditions (Supplementary Figure 5A). Previous studies have also reported an increased leaf protein content, which correlated to increased shoot biomass in chickpea plants that were treated with a consortium of PGPR (Khan et al., 2019).

The measured PGPR-induced remodeling of maize metabolome gravitates also toward priming phenomenology. For instance, TCA cycle intermediates have been shown to accumulate in the pre-challenge stage in response to a priming stimulus, β-aminobutyric acid (BABA) (Gamir et al., 2014); and the decrease in the amino acid levels was reported as a mechanism that sensitizes the plant for primed defense responses (Pastor et al., 2014). Furthermore, PGPR-treatment led to decreased levels of phenylalanine and tyrosine in maize leaves (Figure 2), indicating potentiation of the phenylpropanoid pathway—a central hub for the biosynthesis of defense-related metabolites (Hildebrandt et al., 2015; Parthasarathy et al., 2018). Phenylpropanoid and flavonoid biosynthesis are some of the metabolic pathways involved in PGPR-induced systemic response (ISR) (Garcia-Seco et al., 2015).

**FIGURE 3:**
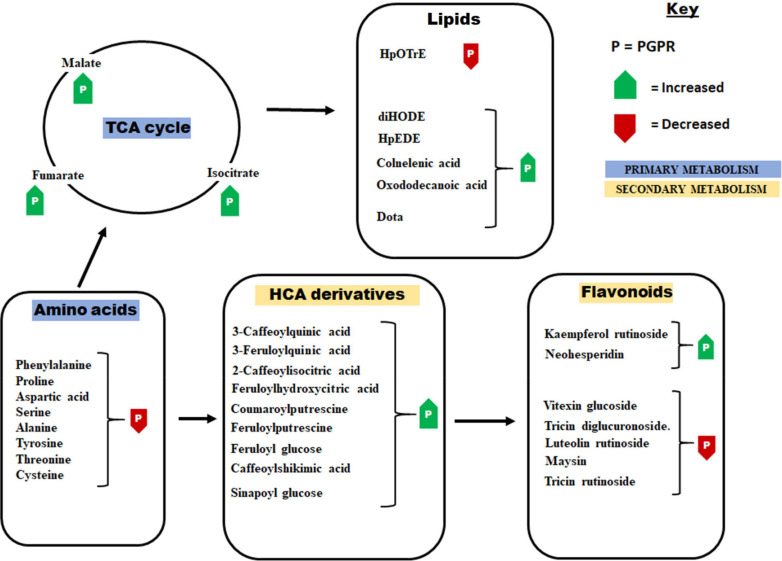
Some of the principal pathways and metabolites that showed differential changes in response to the application of the PGPR-based biostimulant. The PGPR treatment has been shown to induce metabolic readjustment in both primary and secondary metabolism. These metabolic changes were associated with enhanced plant growth and the establishment of a “sensitized” or “ready-to-go” state. HpOTrE, hydroperoxy-octadecatrienoic acid; HpEDE, hydroperoxy-eicosadienoic acid; DiHODE, dihydroxy-octadecadienoate; Dota, dihydroxy-octadecatrienoic acid.

Further metabolic signatures of the PGPR treatment on maize included alterations in ascorbic acid and oxylipins (Figures 2, 3), which point to growth promotion and defense priming. Changes in ascorbic acid levels are associated with various plant growth processes, stress perception and downstream signaling pathways (e.g., hormonal- and redox pathways) (Shapiguzov et al., 2012; Hasanuzzaman et al., 2013; Ben Rejeb et al., 2014; Ivanov, 2014; Akram et al., 2017). Some of the general functions of oxylipins in plants include modifications of chloroplast function, stomatal conductance and root growth inhibition (Genva et al., 2019). Though mechanistically the roles of individual oxylipins are poorly understood, the oxylipin pathway leads to the generation of a signaling phytohormone, jasmonic acid (Wasternack and Feussner, 2018). Thus, the observed changes in the oxylipins correlated to altered signal transduction networks and leading into cellular defense priming ecosystems. To evaluate further the effect of the microbial-based biostimulant on plant signalomics, we quantified some phytohormones (Supplementary Table 2). Differential hormonal changes observed included an increase in ABA, zeatin and SA levels and a decrease of IAA in PGPR-treated non-stressed plants (Supplementary Figure 3). A fine-tuning between SA, ABA and JA have been described as one of the defense preconditioning mechanisms (Pastor et al., 2013), whereas zeatin and IAA are correlated to growth promotion (Ahmed and Hasnain, 2014). As such, PGPR application impacted the hormonal network toward growth promotion and defense priming.

Furthermore, the PGPR induced reprogramming of maize secondary metabolism, as reflected by differential changes in hydroxycinnamic acids (HCAs) derivatives (including HCA-organic acids, HCA-sugars and HCA-amides), and flavonoids (Figures 2, 3). HCA derivatives are involved in a wide spectrum of biological processes including plant development, photo-protection, carbon reservoir, detoxification of ROS and lignin biosynthesis (Leiss et al., 2009; Mandal et al., 2010; Parveen et al., 2013). In this study, the application of the PGPR-based biostimulant led to an increase in the levels of chlorogenic acids (CGAs) (particularly 3-feruloylquinic acid, 3-caffeoylquinic acid, dicaffeoylquinic and coumaroylquinic acid) (Figures 2, 3). The accumulation of chlorogenic acids due to PGPR treatment has been previously reported as one of the growth promotion and stress protection traits in other plants such as chickpea (Singh et al., 2003, 2014). Furthermore, the accumulation of cinnamoyl hydroxycitric acid esters (feruloyl-, caffeoyl-, and coumaroyl hydroxycitric acids) was also observed in PGPR-treated leaves, and this was found to be consistent with the finding of Zhou et al. (2019). Other perturbed HCA compounds included the accumulation of coumaroyl- and feruloyl putrescine in PGPR-based biostimulant treated plants (Figure 2). Several PGPR strains have been shown to enhance the accumulation of HCA-amides in plants for growth promotion and defense priming (Valette et al., 2019). Relative quantitative profiles of flavonoids such as luteolin-rutinoside, maysin, vitexin, neohesperidin, tricin diglucuronoside, tricin rutinoside showed differential changes in response to PGPR treatments (Figures 2, 3). Flavonoids are polyphenols with a wide range of biological functions in plants such as transcriptional and growth regulation, and plant immunity (Mathesius, 2018; Mareya et al., 2019).

Thus, our results reveal that the microbial biostimulant induced a metabolic choreography in maize plants that span a spectrum of primary and secondary metabolic pathways (Figure 3). These changes involve the accumulation of TCA intermediates and ascorbic acid, decreased levels of amino acids, higher levels of phytohormones such as Zea, ABA, SA and decreased level of IAA, higher levels of HCA derivatives and differential changes of lipids and flavonoids. This biostimulant-induced metabolic landscape is a translation of the phenomenology of growth promotion and the preconditioning of the maize plant defenses against adverse environmental conditions. This data-derived hypothetical framework suggests a rewiring of plant metabolism toward the regulation and coordination of cellular (and organismal) events including (i) enhanced energy production, (ii) accelerated protein synthesis, (iii) potentiation of the phenylpropanoid pathway and (iv) regulation of phytohormone biosynthesis, as key mechanisms by which the microbial biostimulant stimulates plant growth and defense priming.

### Global Metabolic Reprogramming Induced by PGPR-Based Biostimulant Application in Maize Plants Under Drought Conditions

**FIGURE 4:**
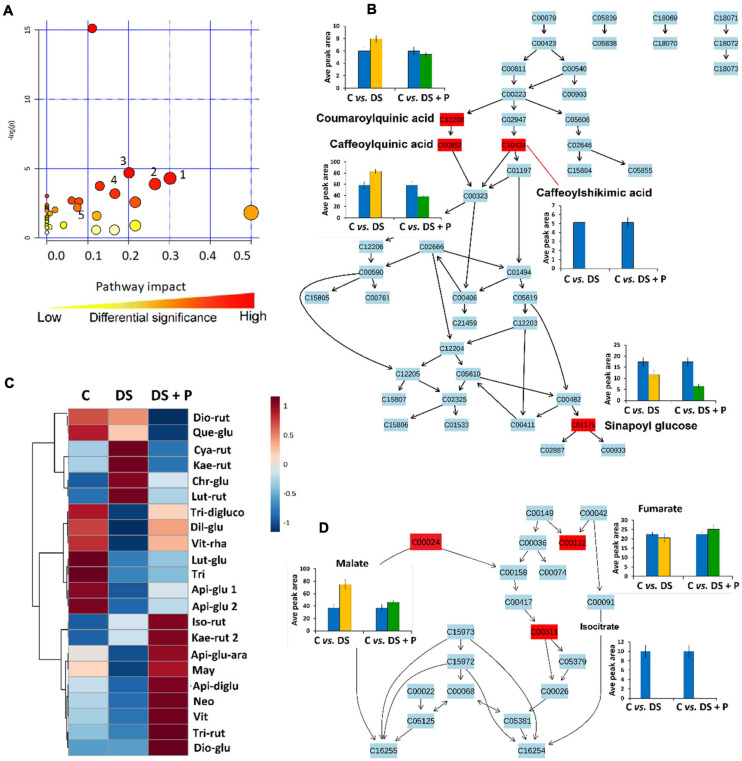
A synopsis of metabolic pathway analysis generated using MetPA, and relative quantification of some altered phenolic compounds and TCA intermediates. **(A)** The graph displaying the “metabolome view” containing all the mapped pathways arranged by *p*-values on the *y*-axis and the pathway impact (differential significance) on the *x*-axis. Pathway impact values refer to the cumulative percentage from the matched metabolite nodes and the maximum importance of each pathway is 1. (**1**) Glycine, alanine and serine metabolism, (**2**) Ascorbate and aldarate metabolism (**3**) Tyrosine metabolism (**4**) Phenylpropanoid biosynthesis and (**5**) Citrate cycle **(B)** Topological characteristics of phenylpropanoid biosynthesis pathway, showing the quantification levels (averaged peak area) of the mapped metabolites. **(C)** A heatmap displaying differential quantitative alterations in the concentrations of the selected flavonoids (refer to Supplementary Table 1 for full names and *p*-values). **(D)** Topological characteristics of the citrate cycle pathway, showing the quantification levels of fumarate, malate and isocitrate under different conditions. C, control (no stress-no PGPR); DS, drought stress-no PGPR; DS + P, drought stress + PGPR treatment.

Global metabolic rewiring was also observed in maize plants treated with the microbial biostimulant under drought conditions. PGPR-induced alterations in the levels of phenolics, lipids, amino acids, hormones and organic acids were interpreted in a global “metabolome view.” Pathway analysis revealed that the most significantly altered pathways in relation to PGPR application under drought stress conditions include: phenylpropanoid biosynthesis, glycine, serine and threonine metabolism, tyrosine metabolism, TCA cycle metabolism, amongst others (Supplementary Table 3 and Figure 4A). These results suggest that PGPR application enhances drought tolerance using highly complex cellular reprogramming characterized by altered metabolism spanning several metabolic pathways which are discussed below.

### PGPR-Based Biostimulants Target Primary Metabolism and Signaling Pathways

The global metabolic reconfiguration induced by PGPR sensitization implicated differential alteration of the TCA cycle (Supplementary Table 3 and Figure 4D). The level of isocitric acid was significantly decreased in both naïve and PGPR-treated plants under drought stress conditions (Figure 4D). On the other hand, the malic acid level was increased in naïve stressed plants *vs.* PGPR-primed stressed plants, while an increased level of fumaric acid was observed in PGPR-primed stressed plants *vs.* naive stressed plants (Figure 4D). These results show that the response of malate and fumaric acid to drought stress is influenced by the observed PGPR-induced accumulation of malic acid and fumaric acid in the pre-challenge phase (Figures 2, 3). Malic acid and fumaric acid are associated with drought adaptation, owing to the osmolytic properties, the ability to regulate intracellular ionic content and stomatal conductance (Rzepka et al., 2009; Suguiyama et al., 2014). Furthermore, these TCA intermediates function as alternative sources of carbon, thus improving the photosynthesis rate and CO_2_ exchange. Well-maintained photosynthesis machinery under drought stress is regarded as one of the characteristics of drought tolerance (Rzepka et al., 2009; Suguiyama et al., 2014).

**FIGURE 5:**
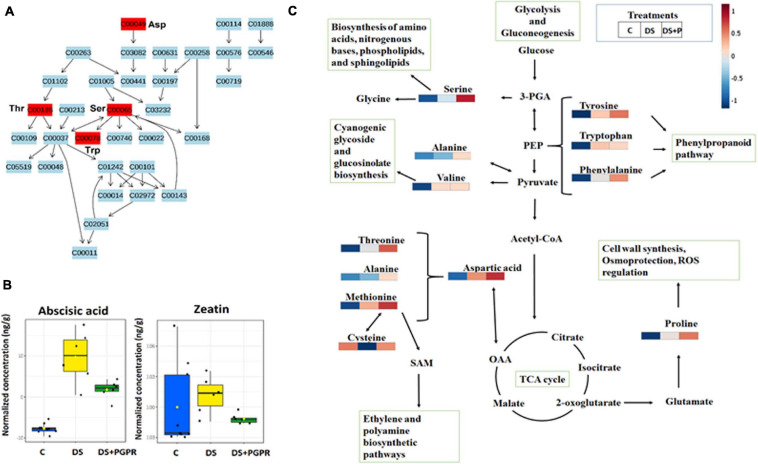
Metabolic pathway analysis and mapping. **(A)** Topology map of glycine, serine and threonine metabolism. **(B)** Boxplots showing the quantification levels of selected phytohormones, abscisic acid and zeatin. **(C)** Amino acids and related metabolic pathways: infographics that show the quantitative changes of amino acids induced by PGPR and their relational properties to other pathways such as citrate (TCA) cycle, phenylpropanoid biosynthesis, polyamine synthesis and glycolysis and gluconeogenesis. C, control: naïve non-stressed; DS, naïve stressed plants; DS + P/PGPR, PGPR- treated stressed plants.

Furthermore, the reconfiguration of primary metabolism in PGPR-based biostimulant-treated stressed plants also implicated a number of significantly impacted pathways such as glycine, serine and threonine metabolism, alanine, aspartic acid and glutamine metabolism, phenylalanine- and tyrosine metabolism (Figure 4). The amino acids spanning these pathways (particularly, serine, threonine, alanine, aspartic acid, phenylalanine and tyrosine) were elevated in PGPR-treated stressed plants compared to naïve stressed plants (Figure 5). The accumulation of some of these amino acids was previously reported in *B. subtilis* B26 treated plants under drought stress (Gagné-bourque et al., 2016). An increased pool of amino acids can be channeled toward energy production and defense-related compounds biosynthesis (Hildebrandt et al., 2015). Moreover, the higher levels of phenylalanine and tyrosine in PGPR-treated stressed plants compared to naïve stressed plants could mean that there is no need for overproduction of defense-related compounds (e.g., phenolics) in the post-challenge phase (Figures 4B,C), especially since they were elevated in the pre-challenge phase *via* microbial-based biostimulant preconditioning (Figure 2). Therefore, this could imply low-cost mechanisms in PGPR-treated stressed plants compared to naïve stressed plants. Generally, increased content of amino acids such as proline, serine, threonine, cysteine, alanine, aspartic acid, phenylalanine and tyrosine associated with drought enhancing mechanisms such as stomatal regulation, osmotic adjustments and oxidative stress protection (Mattioli et al., 2009; Kishor et al., 2015; Wang et al., 2015).

The metabolic reprogramming in naïve and primed (PGPR-treated) plants under drought stress also involved differential alterations in the levels of several phytohormones. These compounds serve as important signaling compounds in response to abiotic and biotic stresses. Furthermore, phytohormones can function synergistically or antagonistically, in crosstalk to mediate a fine-tuning trade-off of resources between the growth and defense response in plants, depending on the circumstances they are faced with (Ma et al., 2019; Tugizimana et al., 2019). To highlight the impact of the microbial-based biostimulant on the phytohormones, we focus on one of the first signaling responses to drought stress, ABA, and a growth hormone, zeatin. The levels of these hormones were higher in naïve stressed plants compared to PGPR-treated stressed plants (Figure 5). The increased level of ABA under drought negatively affects photosynthesis machinery and plant growth (Kim et al., 2010; Lim et al., 2015; Mittler and Blumwald, 2015; Dar et al., 2017). Thus, the observed reduced levels of ABA in PGPR-treated stressed plants combined with the increased level of ABA observed in PGPR-treated plant in the pre-challenge phase (Figure 5B) reflect the “primed state” characterized by the regulation the ABA homeostasis. Furthermore, the differential changes in the level of ABA and zeatin in PGPR-treated stressed plants points to enhanced stomatal regulation, thus improving the photosynthetic efficiency and growth rate under drought conditions (Pavlù et al., 2018). Moreover, these results are supported by higher SPAD readings (reflecting the leaf chlorophyll content) that was measured in the PGPR-treated plants under drought conditions (Supplementary Figure 5B).

### PGPR-Based Biostimulant Significantly Impacted Phenolic Compounds and Lipids Spanning Pathways as Key Mechanisms Facilitating Drought Tolerance

**FIGURE 6:**
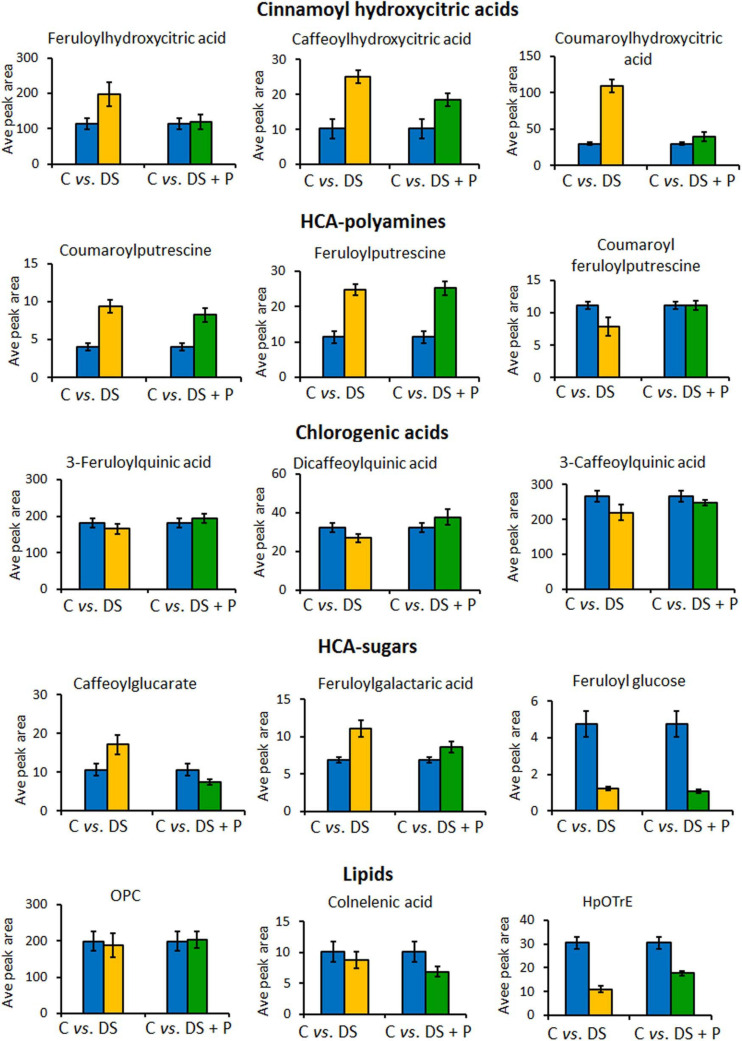
Relative quantitative profiles of some of the phenolic compounds in naïve unstressed plants (C = control), naïve stressed plants (DS = drought stress), and PGPR-primed stressed plants (DS+P = drought stress + PGPR). The bar graphs were generated using averaged integrated peak area (*n* = 9) and the error bars represent the standard deviation. For statistical significance evaluation, the *p*-values are reported in Supplementary Table 1. OPC, oxo-(pentenyl)cyclopentane-octanoic acid; HpOTrE, hydroperoxy-octadecatrienoic acid.

To evaluate the impact of PGPR-based biostimulant on the phenolic compounds spanning pathways under drought stress, we looked at the quantitative profiles of the tentatively annotated phenolics such as HCA derivatives and flavonoids. Although all the chlorogenic acids were upregulated in PGPR-treated non-stressed plants (Figure 2), these compounds were differentially impacted under drought stress. The levels of 5-caffeoylquinic acid and coumaroylquinic acid were decreased in PGPR-primed stressed plants compared to the naïve stressed plants (Figure 4B). Contrastingly, other chlorogenic acids such as feruloylquinic acid and di-caffeoylquinic acid were increased in PGPR-treated plants compared to the naïve stressed plants (Figure 6). These results suggest that the microbial-based biostimulant differentially regulates the metabolic fluxes to facilitate drought tolerance. The roles of chlorogenic acids under drought stress are not clearly defined, however, these HCA compounds are generally associated with radicals scavenging, attributed to their hydroxyl groups (Sharma et al., 2019). Moreover, the increased level of di-caffeoylquinic acid was also observed in globe artichoke under drought stress and was reported to be involved in the strengthening of the cell wall (Nouraei et al., 2018).

Interestingly, caffeoylshikimic acid content was not detected in all drought treated plants and PGPR treatment showed to have no distinguishable effects under drought stress (Figure 4B). In the pre-challenge phase, caffeoylshikimic acid levels were increased in PGPR treated plants (C *vs.* PGPR) (Figure 3). This suggests that the observed caffeoylshikimic acid changes in the pre-challenge phase are only related to growth promotion mediated by this PGPR-based biostimulant but are not related to the defense potentiation effects. On the other hand, the changes in the level of sinapoyl glucose, shown in the post-challenge phase induced by PGPR treatments are related to the alteration observed in the pre-challenge phase. Sinapoyl glucose showed a decrease in both primed and non-primed plants, but the content of this compound was found to be more decreased in PGPR-primed stressed plants than naïve stressed plants (Figure 4B).

Other HCA derivatives that showed differential PGPR-primed responses to drought stress included HCA sugars, cinnamoyl hydroxycitric acid esters and HCA-amides (Figure 6). The levels of caffeoylglucarate and feruloylgalactaric acid were increased in naïve stressed plants compared to PGPR-treated stressed plants (Figure 6). The cinnamoyl hydroxycitric acid esters also followed the same trend as caffeoylglucarate and feruloylgalactaric acid (Figure 5). The application of PGPR formulation seemed to regulate the levels of cinnamoyl hydroxycitric acid esters, counteracting the effects of drought stress (Figure 6). This provides evidence that the preconditioning of cinnamoyl hydroxycitric acid esters may enhance drought resistance, as they are already assumed to have defensive roles against environmental stresses (Zhou et al., 2019). Moreover, polyamines (PAs) and derivatives have been shown to improve drought adaptation by regulating redox homeostasis and stomatal conductance (Agurla et al., 2018; Pál et al., 2019). In our study, the PGPR treatment increased HCA-amides, such as coumaroyl-feruloylputrescine and feruloylputrescine under drought conditions, which could be involved in the above-mentioned metabolic roles (Figure 6).

The other metabolic pathway that was impacted by PGPR treatment is the flavonoid biosynthesis. A range of flavonoids that were differentially altered in the PGPR-primed plants under drought stress included: isomers of kaempferol rutinoside, maysin, neohesperidin, tricin di-glucuronoside, apigenin rutinoside, apigenin glucoside arabinoside, luteolin rutinoside and many more (Supplementary Table 1 and Figure 4D). The enhanced accumulation of flavonoids under drought stress has been reported in several plants’ species (Ma et al., 2014; Rezayian et al., 2018; Gharibi et al., 2019; Sri Sulasmi et al., 2019). Flavonoids have been implicated in redox homeostasis maintenance as well as in stomatal conductance (Zhu and Assmann, 2017). Thus, flavonoids increased by PGPR application could be involved in reducing the ROS in the stomatal guard cells, thus improving stomatal conductance.

Furthermore, alpha-linolenic acid metabolism was revealed as one of the significantly impacted metabolic pathways under PGPR treatment and drought stress (Figure 4A). This pathway was previously reported to be altered under drought stress in thyme plants and has been associated with maintaining the membrane stability under drought stress (Moradi et al., 2017). In our study, differentially altered lipids that are involved in the alpha-linolenic acid pathway include OPC, hydroperoxy-octadecatrienoic acid (HpOTrE), and colnelenic acid. Oxylipins, OPC and HpOTrE, showed an increase in PGPR-primed plants compared to naïve stressed plants, whereas the content of colnelenic acid decreased in PGPR treated stressed plants in comparison to naïve stressed plants (Figure 6).

**FIGURE 7:**
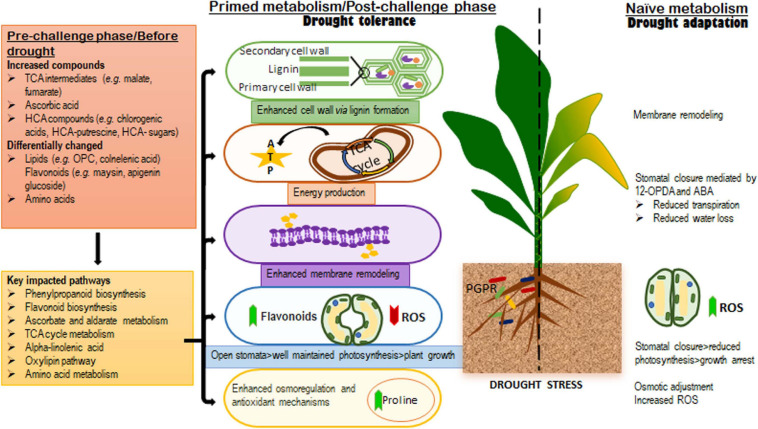
A contextual summary of the results obtained in this study. **The left side of the dotted line** highlights the metabolic and biochemical events in microbial-based biostimulants treated plants; the changes observed in the pre-challenge phase (well-watered conditions), were found to be related to growth promotion and defense preconditioning, whereas, the alterations in the levels of metabolites spanning the key significantly impacted pathways identified in the post-challenge phase (after drought application) were associated with biostimulant-enhanced drought tolerance mechanisms and a “primed” metabolism. **The right side of the dotted** line shows the naïve metabolic responses to drought stress, which are associated with evolutionally developed drought adaptation mechanisms.

In summary, the present study provides key fundamental insights describing a hypothetical framework underlying the effects of microbial-based biostimulants under normal and stress conditions (Figure 7). Metabolic reconfigurations related to the microbial biostimulant-induced growth promotion and priming involves the production of TCA compounds and differential alterations in the levels of amino acids, phenolics and lipids. The main PGPR-mediated mechanisms involved in drought tolerance elucidated in this study, include (i) enhanced cell wall formation, (ii) plasma membrane remodeling, (iii) energy production (iv) enhanced osmoregulation and antioxidant machinery, and (v) stomatal conductance (Figure 7). Contributing to on-going scientific efforts, the knowledge generated from this work lays the foundation for future studies and applications in the biostimulant industry. These prospective investigations may include the identification and understanding of the underlying regulatory networks that define various metabolic fluxes and biochemical machinery in biostimulant-plant-stress interactions. Thus, the biological synopsis derived from these scientific efforts (including the work presented here) would guide PGPR-based biostimulant formulations and contribute to the development of science-based biostimulants for sustainable agriculture.

## Data Availability Statement

The original contributions presented in the study are publicly available. The study design information, LC-MS raw data, analyses and data processing information, and the meta-data have been deposited to the EMBL-EBI metabolomics repository—MetaboLights (Haug et al., 2020) with the identifier MTBLS2566 (www.ebi.ac.uk/metabolights/MTBLS2566).

## Author Contributions

FT, JH, and VM: conceived the project. FT: guided and coordinated the research: LN, PS, NB, and FT: performed the experimental work and analysis and interpretation of the data. LN and FT: writing—original draft preparation. LP, ID, and KB: writing—review and editing. KB, FT, VM, and JH: funding acquisition. All authors have read and agreed to the published version of the manuscript.

## Conflict of Interest

VM, JH, and FT were employed by the company Omnia Group, Ltd. The authors declare that this study received funding from Omnia Group, Ltd. (South Africa). The funder had the following involvement in the study: the planting and collection of samples (plant materials).

## Footnotes

1 https://www.metaboanalyst.ca

2 http://dnp.chemnetbase.com/

3 http://www.chemspider.com/

4 https://www.plantcyc.org/

5 http://www.metaboanalyst.ca
