# Supplementary material for: A Metabolomic Landscape of Maize Plants Treated With a Microbial Biostimulant Under Well-Watered and Drought Conditions
Source: Front Plant Sci. 2021 Jun 3;12:676632. doi: 10.3389/fpls.2021.676632 (PMC8210945; doi:10.3389/fpls.2021.676632)
Supplement: Supplementary file 1 [file Data_Sheet_1.docx]

**Supplementary Materials**

The supplementary figures and tables are provided as additional information to support the main results reported in this study.

**Section S2.1: Imposing drought stress**

The field capacity (FC) and permanent wilting point (PWP) of the soil were determined using the following method. One-pot filled with 17 kg of soil was flooded with water, sealed on top with a clinging wrap and allowed to drain freely from below the pot for 3 days. Thereafter, the mass of the wet soil was weighed (which was taken as the FC). The weighed wet soil was then dried in the oven at 50 °C and weighed; this mass was taken as PWP. The plant available water (PAW) was calculated using the following equations:

1. PAW = FC – PWP
2. 90% PAW = (PAW × 0.9) + PWP
3. 50% PAW = (PAW × 0.5) + PWP


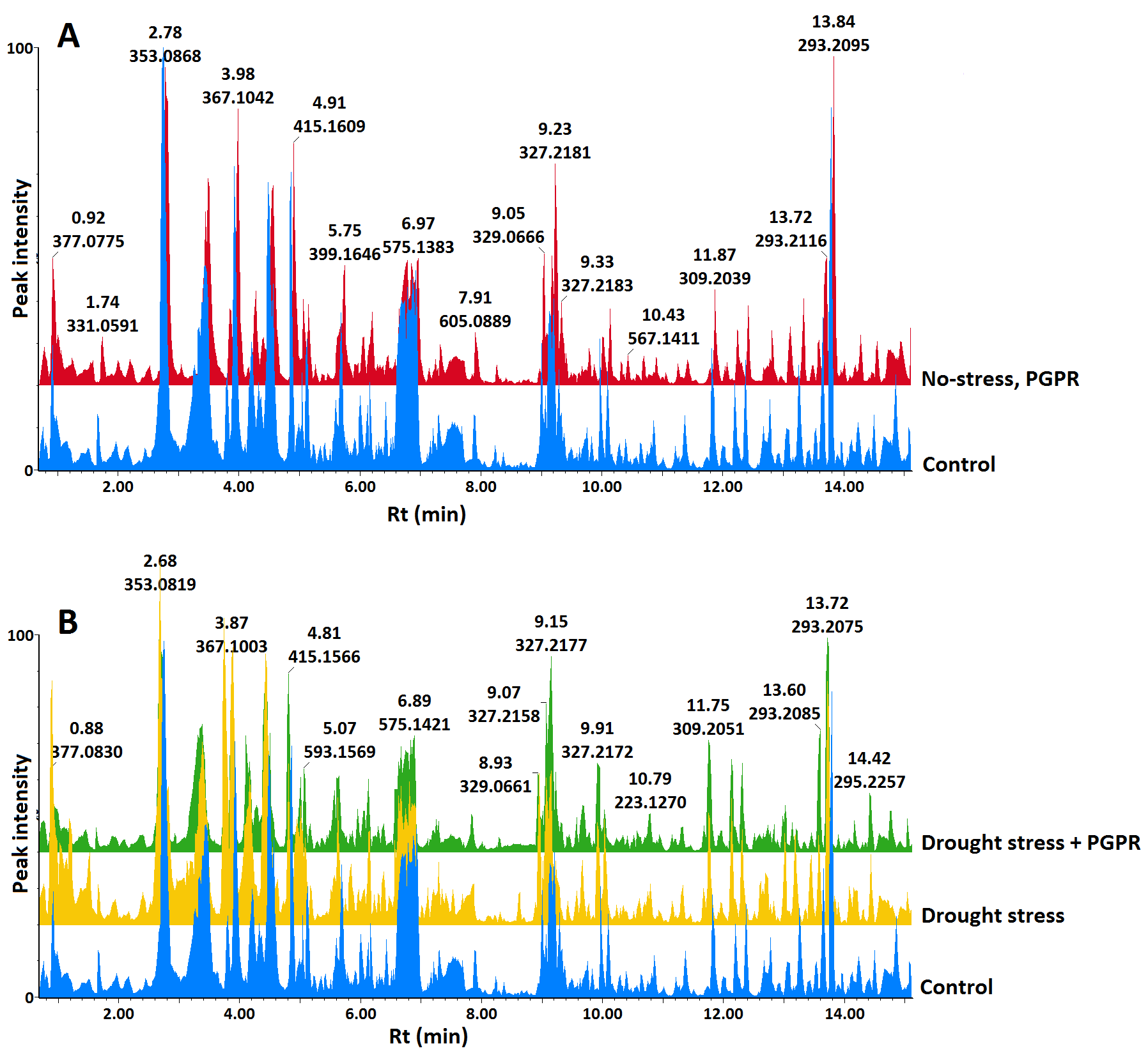


**Supplementary Figure 1.** **Chromatographic analyses of methanolic maize leaf extracts (ESI negative data)**. (**A**) Representative base peak intensity (BPI) chromatograms comparing PGPR-treated to non-treated control under unstressed conditions. (**B**) Representative BPI chromatograms showing a differential peak population due to the application of PGPR formulation under drought stress.


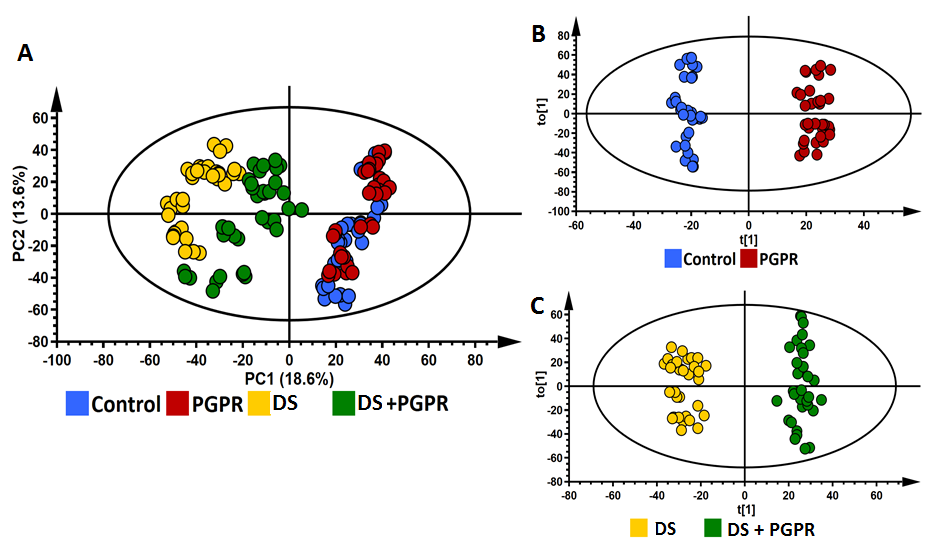


**Supplementary Figure 2. Machine learning analyses of ESI negative data and metabolic classes identified in maize leaves.** (**A**) A PCA score plot of a 10-component model that explains 69.4% of the total variation in Pareto-scaled X data with a 57.1% predictive power (Q^2^). The score plot shows treatment-related grouping. (**B**) OPLS-DA score plot of a 1+1+0 component model with R^2^X (cum) of 33.5%, R^2^Y of 97.4% and Q^2^ (cum) of 96.8%, based on 7-fold cross-validation, and a CV-ANOVA *p-*value of 0. (**C**) OPLS-DA score plot of a 1+2+0 component model with R^2^X (cum) of 41.5%, R^2^Y of 98.9% and Q^2^ (cum) of 97.3%, based on 7-fold cross-validation, and a CV-ANOVA *p-*value of 0. **Abbreviations**: C = control, P = PGPR-treated, DS = drought stress, DS + P = PGPR-treated and drought stress


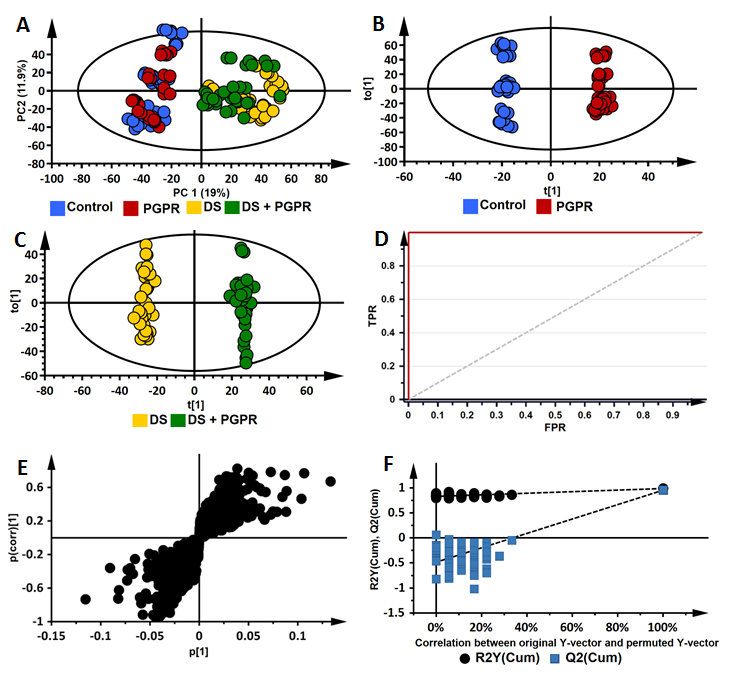


**Supplementary Figure 3.** **Typical infographics from a multivariate machine learning model of ESI positive data**. (**A**) A score plot of an 8-component PCA model that explains 56.6% of the total variation in Pareto-scaled ESI-negative data with a 43.6% predictive power. (**B**) OPLS-DA score plot representing a 1+4+0 component model separating control (C) *vs*. PGPR-treated (P) samples, with R^2^X (cum) of 46.6%, R^2^Y of 99.2% and Q^2^ (cum) of 93.2%, based on 7-fold cross-validation, and a CV-ANOVA *p-*value of 0. (**C**) OPLS-DA score plot representing a 1+3+0 component model separating drought stress (DS) *vs*. PGPR-treated and stressed (DS + PGPR) samples, with R^2^X (cum) of 41.6%, R^2^Y of 98.7% and Q^2^ (cum) of 95.4%, based on 7-fold cross-validation, and a CV-ANOVA *p-*value of 0. (**D**) A representative receiver operator characteristic (ROC) plot summarizing the performance of binary classifier (OPLS-DA). It shows that the computed OPLS-DA is an excellent classifier with 100% sensitivity and 100% specificity as depicted by the ROC curve that passes through the upper left corner. (**E**) A representative OPLS-DA loadings S-plot for the variable section, the discriminant ions are positioned at the ends of ‘S’ shape (variables with high covariation and high correlation). (**F**) A representative permutation plot (n = 100 permutations) with R^2^ intercept (0.0, 0.77) and Q^2^ intercept (0.0, -0.48). **Abbreviations**: C = control, P = PGPR-treated, DS = drought stress, DS + P = PGPR-treated and drought stress

**Supplementary Table 1.** All putatively annotated metabolites in this study. *denotes the abbreviations used in **Supplementary Figure 1.** (Sunburst plot) and **Supplementary Figure 4D.** (heatmap). **Abbreviations:** HCA = hydroxycinnamic acid, C = control, P = PGPR-treated, DS = drought stress, DS + P = PGPR-treated and drought stress.

| No. | Putative annotation | *Abbrev | Rt  (min) | *m/z* | Fragment ions | Adducts | Molecular formula | Class | *p*-values |  |
| --- | --- | --- | --- | --- | --- | --- | --- | --- | --- | --- |
|  |  |  |  |  |  |  |  |  | **C vs P** | **DS vs. DS+P** |
| 1 | Gluconic acid | Gluc | 0.9 | 195.05 | 191,162,108 | [M−H]^−^ | C_6_H_12_O_7_ | Organic acid | 1.97 × 10^−8^ | 0.694 |
| 2 | Hydroxycitric acid | Hyd | 0.92 | 207.02 | 191,189,127 | [M−H] ^−^ | C_6_H_8_O_8_ | Organic acid | 1.43 × 10^−11^ | 4.22 × 10^−8^ |
| 3 | Fumaric acid | Fum | 0.96 | 115.00 | 111 | [M−H] ^−^ | C_4_H_4_O_4_ | Organic acid | 0.335 | 0.439 |
| 4 | Malic acid | Mal | 0.96 | 133.01 | 115 | [M−H] ^−^ | C_4_H_6_O_5_ | Organic acid | 0.408 | 2.59 × 10^−10^ |
| 5 | Isocitric acid | Iso | 1.22 | 191.02 | 173,129,111,85 | [M−H] ^−^ | C_6_H_8_O_7_ | Organic acid | 3.03 × 10^−3^ | 5.97 × 10^−12^ |
| 6 | Isoleucine | Ile | 1.42 | 132.10 | 86 | [M+H]^+^ | C_6_H_13_NO_2_ | Amino acid | 3.68 × 10^−2^ | 6.30 × 10^−3^ |
| 7 | Caffeoylglucarate 1 | Caf-glu 1 | 1.52 | 371.06 | 209,173,129,85 | [M−H] ^−^ | C_15_H_16_O_11_ | HCA-sugar | 1.72 × 10^−3^ | 2.89 × 10^−23^ |
| 8 | Galloyl-hexoside | Gal | 1.68 | 331.06 | 168,125 | [M−H] ^−^ | C_13_H_16_O_10_ | Phenolic compound | 0.183 | 0.358 |
| 9 | Caffeoylglucarate 2 | Caf-glu 2 | 1.75 | 371.06 | 209,173,143,129,85 | [M−H] ^−^ | C_15_H_16_O_11_ | HCA-sugar | 6.28 × 10^−3^ | 8.71 × 10^−5^ |
| 10 | Caffeoylglucarate 3 | Caf-glu 3 | 1.93 | 371.06 | 209,177,147,129,86 | [M˗H] ^−^ | C_15_H_16_O_12_ | HCA-sugar | 0.407 | 9.64 × 10^−16^ |
| 11 | Phenylalanine | Phe | 1.97 | 166.08 | 120,103 | [M+H]^+^ | C_9_H_11_NO_2_ | Amino acids | 0.105 | 1.29 × 10^−8^ |
| 12 | Caffeoylglucarate 4 | Caf-glu 4 | 1.98 | 371.06 | 209,173,164,129 | [M−H] ^−^ | C_15_H_16_O_11_ | HCA-sugar | 0.379 | 0.036 |
| 14 | Caffeoylputrescine | Caf-put | 2.38 | 251.14 | 163,86 | [M+H]^+^ | C_13_H_18_N_2_O_3_ | HCA-amide | 0.671 | 0.447 |
| 15 | DIBOA-glucoside | DIBOA-glu | 2.43 | 342.08 | 180,153 | [M−H] ^−^ | C_14_H_17_NO_9_ | Benzoxaxinoid | 0.440 | 0.077 |
| 16 | 3-Caffeoylquinic acid | 3-CafQA | 2.72 | 353.86 | 191,179,173,135 | [M−H] ^−^ | C_16_H_18_O_9_ | Chlorogenic acid | 0.7420 | 3.49 × 10^−5^ |
| 17 | Tryptophan | Typ | 2.97 | 205.09 | 188 | [M+H]^+^ | C_11_H_12_N_2_O_2_ | Amino acids | 0.483 | 4.44 × 10^−4^ |
| 18 | Coumaroylputrescine | Cou-put | 3.01 | 235.14 | 218,188,147,146,127 | [M+H]^+^ | C1_3_H_18_N_2_O_2_ | HCA-amide | 3.88 × 10^−3^ | 5.46 × 10^−7^ |
| 19 | Caffeoyltyptamine | Caf-typ | 3.07 | 323.17 | 163 | [M+H]^+^ | C_19_H_18_N_2_O_3_ | HCA-amide | 6.47 × 10^−2^ | 5.55 × 10^−8^ |
| 20 | Feruloylgalactaric acid | Fer-gal | 3.08 | 385.07 | 223,191,193,189 | [M−H] ^−^ | C_16_H_18_O_11_ | HCA-sugar | 2.51 × 10^−3^ | 7.46 × 10^−5^ |
| 21 | Caffeoylhydroxycitric acid 1 | Caf-hyd 1 | 3.38 | 369.04 | 207, 189, 127 | [M−H] ^−^ | C_15_H_14_O_11_ | HCA-organic acid | 0.259 | 1.44 × 10^−4^ |
| 22 | Hydroxycoumarin | Hyd-cou | 3.4 | 163.04 | - | [M+H]^+^ | C_9_H_6_O_3_ | HCA | 1.88 × 10^−34^ | 0.261 |
| 23 | Caffeoylhydroxycitric acid 2 | Caf-hyd 2 | 3.46 | 369.98 | 207,189, 127 | [M−H] ^−^ | C_15_H_14_O_11_ | HCA-organic acid | 7.06 × 10^−2^ | 1.44 × 10^−8^ |
| 24 | Feruloylputrescine | Fer-put | 3.48 | 265.15 | 177,117,163,89 | [M+H]^+^ | C_14_H_20_N_2_O_3_ | HCA-amide | 1.16 × 10^−5^ | 0.115 |
| 25 | 5-Coumaroylquinic acid | 5-CouQA | 3.51 | 337.10 | 191,163 | [M−H] ^−^ | C_16_H_17_O_8_ | Chlorogenic acid | 4.30 × 10^−2^ | 1.40 × 10^−2^ |
| 26 | Glucosyl-apigenin | Glu-api | 3.67 | 431.15 | 269 | [M−H] ^−^ | C_21_H_20_O_10_ | Flavonoid | 0.778 | 4.59 × 10^−9^ |
| 27 | 5-Caffeoylquinic acid | 5-CafQA | 3.8 | 353.08 | 191 | [M−H] ^−^ | C_16_H_18_O_9_ | Chlorogenic acid | 0.880 | 1.05 × 10^−4^ |
| 28 | 3-Feruloylquinic acid I | 3-FerQA | 3.91 | 367.10 | 193,173,134 | [M−H] ^−^ | C_17_H_20_O_9_ | Chlorogenic acid | 0.956 | 0.140 |
| 29 | Coumaroylhydroxycitric acid | Cou-hyd | 4.11 | 353.05 | 189,127,99,83 | [M−H] ^−^ | C_15_H_14_O_10_ | HCA-organic acid | 6.30 × 10^−3^ | 6.12 × 10^−11^ |
| 30 | Coumarin | Coum | 4.19 | 147.04 | 91 | [M+H]^+^ | C_9_H_6_O_2_ | HCA | 0.7544 | 2.70 × 10^−2^ |
| 31 | Sinapoyl glucose | Sin-glu | 4.27 | 385.11 | 223,205,179 | [M−H] ^−^ | C_17_H_22_O_10_ | HCA-sugar | 6.10 × 10^−2^ | 2.04 × 10^−3^ |
| 32 | Feruloyl glucose | Fer-glu | 4.28 | 355.10 | 193 | [M−H] ^−^ | C_16_H_20_O_9_ | HCA-sugar | 0.108 | 1.75 × 10^−12^ |
| 33 | Coumaroyl feruloylputrescine | Cou-fer-put | 4.3 | 411.22 | 259,208,177,147 | [M+H]^+^ | C_23_H_26_N_2_O_5_ | HCA-amide | 0.195 | 0.633 |
| 34 | Sinapinic acid glucuronide | Sin-glucu | 4.35 | 399.09 | 237,229,75 | [M−H] ^−^ | C_17_H_20_O_11_ | HCA-sugar | 1.68 × 10^−4^ | 0.612 |
| 35 | Caffeoylisocitric acid | Caf-iso | 4.4 | 353.05 | 191,173,155,111 | [M−H] ^−^ | C_16_H_18_O_9_ | HCA-organic acid | 0.821 | 0.229 |
| 36 | Ascorbic acid | Asc | 4.45 | 177.05 | 145 | [M+H]^+^ | C_6_H_8_O_6_ | Organic acid | 3.20 × 10^−6^ | 6.40 × 10^−2^ |
| 37 | 2-Feruloylhydroxycitric acid 1 | Fer-hyd | 4.47 | 383.06 | 189,127,33,83 | [M−H] ^−^ | C_16_H_16_O_11_ | HCA-organic acid | 9.43 × 10^−10^ | 2.223 × 10^−11^ |
| 38 | DIMBOA | DIMBOA | 4.57 | 212.05 | 193,177,166 | [M+H]^+^ | C_9_H_9_NO_5_ | Benzoxazinoid | 0.868 | 2.80 × 10^−2^ |
| 39 | Dicaffeoylquinic acid | DicafQA | 4.58 | 515.12 | 353,191,179, | [M−H] ^−^ | C_25_H_23_O_12_ | Chlorogenic acid | 0.430 | 2.80 × 10^−4^ |
| 40 | 5-Coumaroylquinic acid | 5-CouQA | 4.64 | 337.09 | 191,163 | [M−H] ^−^ | C_16_H_17_O_8_ | Chlorogenic acid | 4.34 × 10^−2^ | 1.44 × 10^−2^ |
| 41 | Caffeoylshikimic acid | Caf-shi | 4.79 | 335.08 | 191, 179, 135 | [M−H] ^−^ | C_16_H_16_O_8_ | HCA-organic acid | 3.71 × 10^−9^ | 1.25 × 10^−23^ |
| 42 | 2-Feruloylhydroxycitric acid 2 | Fer-hyd | 4.93 | 383.06 | 189,127,33,83 | [M−H] ^−^ | C_16_H_16_O_11_ | HCA-organic acid | 0.839 | 0.305 |
| 43 | 3-Feruloylquinic acid 2 | FerQA 2 | 4.97 | 367.10 | 193,173,134 | [M−H] ^−^ | C_17_H_20_O_9_ | HCA-organic acid | 0.588 | 9.42 × 10^−3^ |
| 44 | Apigenin glucoside arabinoside | Api-glu-ara | 5.04 | 563.14 | 473,443,383,353 | [M−H] ^−^ | C_26_H_28_O_14_ | Flavonoid | 0.433 | 4.4 × 10^−13^ |
| 45 | Vitexin glucoside | Vit-glu | 5.1 | 593.15 | 473,447,429 | [M−H] ^−^ | C_27_H_30_O_15_ | Flavonoid | 0.170 | 4.56 × 10^−9^ |
| 46 | Cyanidin rutinoside | Cya-rut | 5.12 | 595.17 | 449,300,299,287 | [M+H]^+^ | C_27_H_31_O_15_^+^ | Flavonoid | 0.166 | 9.09 × 10^−3^ |
| 47 | Chrysoeriol glucuronide | Chr-glucu | 5.2 | 477.31 | 301 | [M+H]^+^ | C_22_H_20_O_12_ | Flavonoid | 2.41 × 10^−7^ | 0.901 |
| 48 | Dimethoxycinnamic acid | Dimet | 5.36 | 209.15 | 177,163,149 | [M+H]^+^ | C_11_H_12_O_4_ | HCA | 4.30 × 10^−2^ | 0.939 |
| 49 | Tricin diglucuronoside. | Tri-diglucu | 5.57 | 681.13 | 533,351 | [M−H] ^−^ | C_29_H_30_O_19_ | Flavonoid | 0.136 | 1.75 × 10^−20^ |
| 50 | Isoquercetin | Isoque | 5.64 | 465.10 | 303 | [M+H]^+^ | C_21_H_20_O_12_ | Flavonoid | 0.226 | 1.60 × 10^−2^ |
| 51 | Neohesperidin | Neo | 5.65 | 609.14 | 301,300,293 | [M−H] ^−^ | C_28_H_34_O_15_ | Flavonoid | 2.78 × 10^−22^ | 4.39 × 10^−2^ |
| 52 | Vitexin | Vit | 5.71 | 431.09 | 341,311,283,269 | [M−H] ^−^ | C_21_H_20_O_10_ | Flavonoid | 0.146 | 4.59 × 10^−7^ |
| 53 | Dillenetin glucoside-glucuronide | Dil-glu-glucu | 5.8 | 667.15 | 329 | [M−H] ^−^ | C_29_H_32_O_18_ | Flavonoid | 0.229 | 9.99 × 10^−5^ |
| 54 | Quercetin glucoside | Que-glu | 5.85 | 463.09 | 301, 300 | [M−H] ^−^ | C_21_H_20_O_12_ | Flavonoid | 0.376 | 0.991 |
| 55 | Luteolin rutinoside | Lut-rut | 6.15 | 595.16 | 287,331,449 | [M+H]^+^ | C_27_H_31_O_15_ | Flavonoid | 2.22 × 10^−5^ | 2.18 × 10^−10^ |
| 56 | Isorhamnetin rutinoside | Iso-rut | 6.25 | 623.16 | 315,314,300 | [M−H] ^−^ | C_28_H_32_O_16_ | Flavonoid | 0.659 | 0.537 |
| 57 | Tricin rutinoside I | Tri-rut 1 | 6.3 | 637.14 | 329,315 | [M−H] ^−^ | C_29_H_34_O_16_ | Flavonoid | 3.8 × 10^−2^ | 1.15 × 10^−6^ |
| 58 | Diosmetin glucoside | Dio-glu | 6.36 | 461.11 | 299,285,284 | [M−H] ^−^ | C_22_H_22_O_11_ | Flavonoid | 0.124 | 0.342 |
| 59 | Luteolin glucoside | Lut-glu | 6.37 | 447.09 | 285,284 | [M−H] ^−^ | C_21_H_20_O_11_ | Flavonoid | 1.26 × 10^−7^ | 3.28 × 10^−6^ |
| 60 | Vitexin rhamnoside 1 | Vit-rha 1 | 6.41 | 577.15 | 473,437,431,413,357 | [M−H] ^−^ | C_27_H_30_O_14_ | Flavonoid | 0.459 | 0.134 |
| 61 | Kaempferol rutinoside 1 | Kae-rut 1 | 6.59 | 595.16 | 577, 449,432, 413,287 | [M+H]^+^ | C_27_H_30_O_15_ | Flavonoid | 0.771 | 0.373 |
| 62 | Kaempferol rutinoside 2 | Kae-rut 2 | 6.67 | 593.15 | 575,473,411,337,298,285 | [M−H] ^−^ | C_27_H_28_O_14_ | Flavonoid | 0.773 | 1.20 × 10^−3^ |
| 63 | Maysin | May | 6.83 | 575.14 | 473,411,337,298,285 | [M−H] ^−^ | C_27_H_28_O_14_ | Flavonoid | 0.414 | 1.44 × 10^−17^ |
| 64 | Kaempferol rutinoside 3 | Kae-rut | 7.17 | 594.19 | 577, 449,432, 413,287 | [M+H]^+^ | C_27_H_30_O_15_ | Flavonoid | 0.440 | 7.70 × 10^−2^ |
| 65 | Vitexin rhamnoside 2 | Vit-rha 2 | 7.17 | 577.19 | 503,473,415,353 | [M−H] ^−^ | C_27_H_30_O_14_ | Flavonoid | 0.554 | 3.42 × 10^−4^ |
| 66 | Diosmetin rutinoside | Dio-rut | 7.42 | 607.16 | 299,284 | [M−H] ^−^ | C_28_H_32_O_15_ | Flavonoid | 0.527 | 5.64 × 10^−3^ |
| 67 | Tricin | Tri | 8.98 | 329.06 | 315,314,299 | [M−H] ^−^ | C_17_H_14_O_7_ | Flavonoid | 1.01 × 10^−4^ | 0.115 |
| 68 | Coumaroyl-feruloylglycerol | Cou-fer-gly | 9.17 | 413.12 | 235,193,163 | [M−H] ^−^ | C_22_H_22_O_8_ | HCA-sugar | 0.421 | 1.7 × 10^−9^ |
| 69 | Tricin-O-(erythro-beta-guaiacylglyceryl) ether | Tri-ether | 9.31 | 527.15 | 331 | [M+H]^+^ | C_27_H_26_O_11_ | Flavonoid | 1.00 × 10^−2^ | 8.73 × 10^−4^ |
| 70 | Traumatic acid | Tra | 9.58 | 227.15 | 183,165,160 | [M−H] ^−^ | C_12_H_20_O_4_ | Lipid | 6.58 × 10^−3^ | 4.25 × 10^−7^ |
| 71 | Oxododecanoic acid | Oxo | 9.71 | 213.15 | 209,195,183,171 | [M−H] ^−^ | C_12_H_22_O_3_ | Lipid | 0.729 | 2.08 × 10^−4^ |
| 72 | Dioxooctadecadienoic acid | Diox | 10.8 | 308.20 | 289, 223 | [M−H] ^−^ | C_18_H_28_O_4_ | Lipid | 0.356 | 4.30 × 10^−2^ |
| 73 | Oxo-dihydroxy-octadecadienoic acid 1 | DiHODE 1 | 11.01 | 325.20 | 307,227,209,153 | [M−H] ^−^ | C_18_H_30_O_5_ | Lipid | 3.48 × 10^−4^ | 0.969 |
| 74 | Epoxy-hydroxy-octadecenoic acid 1 | Epox 1 | 11.18 | 311.18 | 211,201 | [M−H] ^−^ | C_18_H_32_O_4_ | Lipid | 4.46 × 10^−\3^ | 5.57 × 10^−7^ |
| 75 | Hydroperoxy-eicosadienoic acid | HpEDE | 11.32 | 339.21 | 307,289,245 | [M−H] ^−^ | C_20_H_36_O_4_ | Lipid | 0.482 | 3.00 × 10^−\3^ |
| 76 | Epoxy-hydroxy-octadecenoic acid 2 | Epox 2 | 11.67 | 311.22 | 211,201 | [M−H] ^−^ | C_18_H_32_O_4_ | Lipid | 5.06 × 10^−8^ | 0.902 |
| 77 | Dihydroxy-octadecadienoic acid | DiHODE 2 | 11.97 | 311.22 | 309,291,211 | [M−H] ^−^ | C_18_H_32_O_4_ | Lipid | 3.20 × 10^−6^ | 6.45 × 10^−2^ |
| 78 | Dihydroxy-octadecatrienoate | Dota | 12.28 | 309.20 | 197,171 | [M−H] ^−^ | C_18_H_30_O_4_ | Lipid | 5.87 × 10^−3^ | 0.996 |
| 79 | Hydroperoxy-octadecatrienoic acid | HpOTrE 1 | 12.68 | 309.20 | 291 | [M−H] ^−^ | C_18_H_30_O_4_ | Lipid | 4.30 × 10^−2^ | 0.939 |
| 80 | Colnelenic acid | Col | 13.17 | 291.19 | 273,247,199 | [M−H] ^−^ | C_18_H_28_O_3_ | Lipid | 3.88 × 10^−3^ | 5.46 × 10^−7^ |
| 81 | Oxo-(pentenyl)cyclopentaneoctanoic acid | OPC | 13.75 | 293.21 | 279,223 | [M−H] ^−^ | C_18_H_30_O_3_ | Lipid | 8.50 × 10^−2^ | 0.218 |
| 82 | Hydroperoxy-octadecatrienoic acid | HpOTrE 2 | 13.76 | 309.21 | 291 | [M−H] ^−^ | C_18_H_30_O_4_ | Lipid | 0.310 | 3.48 × 10^−3^ |
| 83 | Palmitoylglycerol-phosphate | Pal-pho | 14.45 | 431.22 | 295,277,195,152 | [M−H−Na] ^−^ | C_19_H_39_O_7_P | Lipid | 0.588 | 0.782 |

**Supplementary Table 2.** The **MRM-MS method** developed and optimized by direct infusion and the collision energy (CE) optimized for transitions of each compound using the MRM optimization method tool.

| **Compound name** | **Rt (min)** | **Ion mode** | ***m/z*** | **Transition** | **CE (eV)** | **Quadrupole 1**  **(Q1), V** | **Quadrupole 3**  **(Q3), V** | **Dwell time (msec)** |
| --- | --- | --- | --- | --- | --- | --- | --- | --- |
| **Amino acids** |  |  |  |  |  |  |  |  |
| Proline (Pro) | 1.503 | [M+H]^+^ | 116.20 | 116.20>70.15  116.20>43.10 | -18.0  -28.0 | -14.0  -13.0 | -11.0  -15.0 | 17.0  17.0 |
| Cysteine (Cys) | 1.317 | [M+H]^+^ | 241.20 | 151.90 | -14.0 | -12.0 | -15.0 | 37.0 |
| Serine (Ser) | 1.341 | [M+H]^+^ | 106.20 | 106.20>59.95  106.20>88.10 | -13.0  -13.0 | -12.0  -12.0 | -10.0  -18.0 | 17.0  17.0 |
| Alanine (Ala) | 1.406 | [M+H]^+^ | 90.20 | 90.20>44.05  90.20>44.90 | -13.0  -30.0 | -10.0  -18.0 | -15.0  -16.0 | 17.0  17.0 |
| Threonine (Thr) | 1.409 | [M+H]^+^ | 120.20 | 120.20>56.05 120.20>74.10 | -16.0  -12.0 | -13.0  -13.0 | -20.0  -28.0 | 17.0  17.0 |
| Aspartic acid (Asp) | 1.410 | [M+H]^+^ | 134.05 | 134.05>74.10 | -15.0 | -10.0 | -13.0 | 37.0 |
| Valine (Val) | 1.730 | [M+H]^+^ | 118.20 | 118.20>72.10  118.20> 55.05 | -12.0  -23.0 | -14.0  -14.0 | -12.0  -23.0 | 17.0  17.0 |
| Methionine (Met) | 2.142 | [M+H]^+^ | 150.20 | 150.20>60.90  150.20>56.10 | -17.0  -24.0 | -10.0  -11.0 | -20.0  -24.0 | 17.0  17.0 |
| Tyrosine (Tyr) | 3.292 | [M+H]^+^ | 182.00 | 182.0>136.10 | -14.0 | -13.0 | -24.0 | 37.0 |
| Phenylalanine (Phe) | 5.929 | [M+H]^+^ | 166.00 | 166.00>120.10 | -14.0 | -12.0 | -21.0 | 131.0 |
| Tryptophan (Trp) | 6.774 | [M+H]^+^ | 205.20 | 205.20>188.05 205.20>146.10 | -11.0  -17.0 | -14.0  -14.0 | -19.0  -14.0 | 64.0  64.0 |
| **Hormones** |  |  |  |  |  |  |  |  |
| Abscisic acid (ABA) | 7.917 | [M+H]^+^ | 265.10 | 265.10>247.20  265.10>229.30  265.10>201.15 | -8.0  -10.0  -13.0 | -20.0  -13.0  -13.0 | -20.0  -24.0  -21.0 | 65.6  65.6  65.6 |
| Indole-3-acetic acid (IAA) | 26.81 | [M+H]^+^ | 176.10 | 176.10>130.10  176.10>77.20  176.10>103.10 | -15.0  -43.0  -30.0 | -20.0  -12.0  -12.0 | -20.0  -20.0  -22.0 | 65.6  65.6  65.6 |
| Zeatin (Zea) | 12.984 | [M+H]^+^ | 220.15 | 220.15>202.05  220.15>136.00 220.15>119.00 | -19.0  -24.0  -34.0 | -10.0  -11.0  -10.0 | -19.0  -24.0  -11.0 | 100.0  100.0  100.0 |
| Salicylic acid (SA) | 22.598 | [M-H]^-^ | 137.00 | 137.00>92.95  137.00>65.00  137.00>75.05 | 15.0  28.0  32.0 | 20.0  14.0  14.0 | 20.0  10.0  27.0 | 65.6  65.6  65.6 |
| Amino-cyclopropane carboxylic acid (ACC) | 1.58 | [M+H]^+^ | 101.60 | 101.60>56.20  101.60>28.15  101.60>30.20 | -14.0  -23.0  -37.0 | -18.0  -18.0  -18.0 | -21.0  -10.0  -30.0 | 65.6  65.6  65.6 |
| Indole-3-carboxaldehyde* (I3A) | 16.602 | [M+H]^+^ | 146.05 | 146.05 | -25.0 | - | - | 100.0 |
| Indole-3-carboxylic acid* (I3CA) | 22.002 | [M+H]^+^ | 161.95 | 161.95 | -15.0 | - | - | 100.0 |

* These compounds did not fragment, thus quantified using single ion monitoring (SIM)


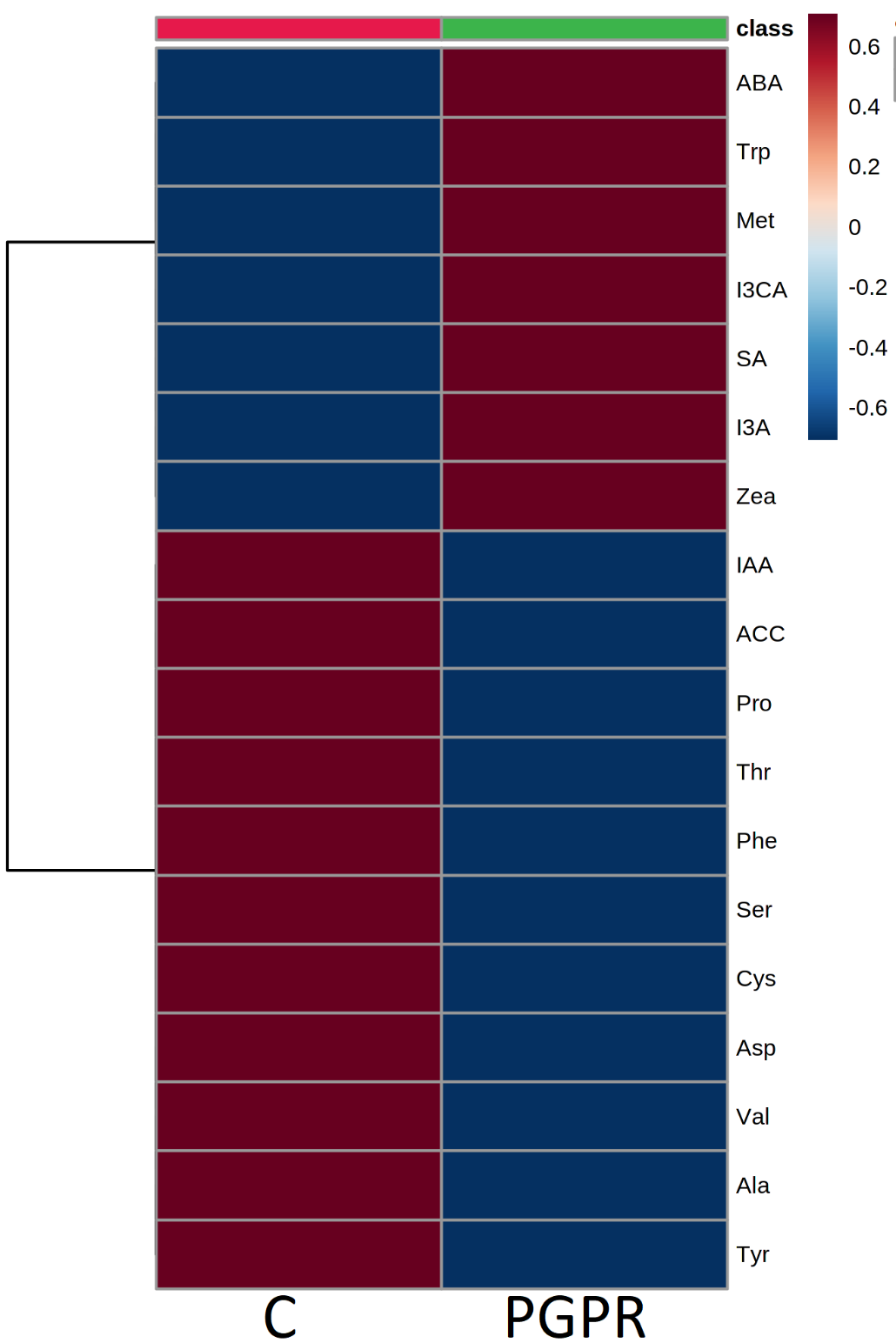


**Supplementary Figure 4.** Heatmap showing quantitative changes in the phytohormone levels in non-treated plants (control) and PGPR-treated plants. **Abbreviations**: C = control: naïve non-stressed plants, PGPR = biostimulant treated non-stressed plants, **amino acids** (Tyr = tyrosine, Phe = phenylalanine, Trp = tryptophan, Ala = alanine, Val = valine, Pro = proline, Asp = aspartic acid, Thr = threonine, Ser = serine, Cys = cysteine, Met = methionine,) and **hormones** (ABA = abscisic acid, Zea = zeatin, SA = salicylic acid, IAA = indole acetic acid, ACC = 1-Aminocyclopropane-1-carboxylic acid, I3CA = indole carboxylic acid, I3A = indole carboxaldehyde).

**Supplementary Table 3.** **Significant metabolic pathways altered in PGPR primed- and non-primed maize plants responding to drought stress, generated from Metabolomics Pathway Analysis (MetPA).** The statistical *p values* from enrichment analysis are further adjusted for multiple analyses to account for numerous metabolic pathways that are tested all at once. The **Raw *p*** is the original *p-value* calculated from the enrichment analysis and the **Holm *p*** is the *p-value* adjusted by the Holm-Bonferroni method. **Hits** indicated the number of matched metabolites in the pathway from uploaded data. The **Impact** is the pathway impact value calculated from pathway topology analysis.

| No. | Pathway name | Hits | Raw p | Holm adjust | Impact |
| --- | --- | --- | --- | --- | --- |
| 1 | Isoquinoline alkaloid biosynthesis | 1 | 0.16 | 1 | 0.50 |
| 2 | Glycine, serine and threonine metabolism | 4 | 0.01 | 1 | 0.30 |
| 3 | Stilbenoid, diarylheptanoid and gingerol biosynthesis | 2 | 0.02 | 1 | 0.26 |
| 4 | Ascorbate and aldarate metabolism | 1 | 0.41 | 1 | 0.22 |
| 5 | Tyrosine metabolism | 2 | 0.08 | 1 | 0.22 |
| 6 | Cysteine and methionine metabolism | 5 | 0.01 | 0.87 | 0.20 |
| 7 | Phenylpropanoid biosynthesis | 4 | 0.04 | 1 | 0.17 |
| 8 | alpha-Linolenic acid metabolism | 1 | 0.56 | 1 | 0.17 |
| 9 | Alanine, aspartate and glutamate metabolism | 3 | 0.02 | 1 | 0.13 |
| 10 | Glyoxylate and dicarboxylate metabolism | 2 | 0.20 | 1 | 0.12 |
| 11 | Tryptophan metabolism | 1 | 0.56 | 1 | 0.12 |
| 12 | Aminoacyl-tRNA biosynthesis | 10 | 2.69 × 10^−7^ | 0.00 | 0.11 |
| 13 | Arginine and proline metabolism | 3 | 0.07 | 1 | 0.08 |
| 14 | Citrate cycle (TCA cycle) | 2 | 0.11 | 1 | 0.07 |
| 15 | Sulfur metabolism | 2 | 0.07 | 1 | 0.06 |
| 16 | Flavonoid biosynthesis | 2 | 0.40 | 1 | 0.04 |


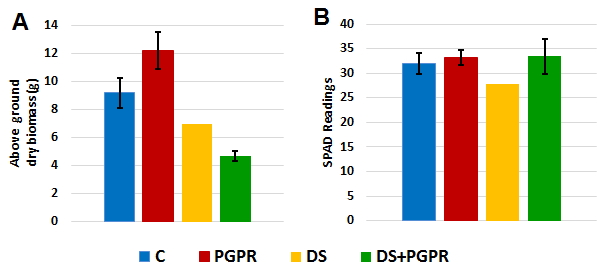


**Supplementary Figure 5**. (**A**) Agronomic (above ground dry biomass) and (**B**) physiological (SPAD; chlorophyll content) measurements. The Minolta SPAD meter was used to take readings from 4 youngest matured leaves and the average was recorded. **Abbreviations**: C = control, PGPR = PGPR-treated, DS = drought stress, DS + PGPR = PGPR-treated and drought stress.
